# Leisure-time Physical Activity and Sedentary Behaviour in Older People: The Influence of Sport Involvement on Behaviour Patterns in Later Life

**DOI:** 10.3934/publichealth.2017.2.171

**Published:** 2017-05-04

**Authors:** Amy M. Gayman, Jessica Fraser-Thomas, Jamie E. L. Spinney, Rachael C. Stone, Joseph Baker

**Affiliations:** 1. School of Kinesiology and Health Science, York University, 4700 Keele St. Toronto, ON, M3J 1P3, Canada; 2. Department of Geography, South Dakota State University, Wecota Annex414, Brookings, SD, 57007, USA

**Keywords:** older adults, sport, sedentary behaviour, physical activity, time use, leisure-time

## Abstract

Given the dramatic demographic change underway in most industrialized nations, the health of older adults is a major concern, particularly given the prevalence of sedentary behaviours and physical inactivity among ageing populations. Researchers have suggested sport participation in later life promotes other health-related behaviours, however, these relationships are poorly understood. It is possible for individuals to be classified as sufficiently active and still spend most of their day involved in sedentary pursuits. Moreover, there is little information on older sport participants' use of time compared to leisurely active or inactive peers and whether type of physical activity involvement is associated with differences in older adults' behaviour patterns. With this in mind, data from 1,723 respondents (65 years and older) who completed the sport module of the 2010 Canadian General Social Survey–Time Use were used to investigate the influence of physical activity involvement (competitive sport vs. non-competitive sport vs. physically active leisure vs. inactivity) on time spent in leisure-time physical activity and sedentary behaviours. Results indicated that competitive sport participants spent less time engaging in sedentary behaviours compared to the physically active leisure or inactive respondents; however, sport participants (both competitive and non-competitive) also spent less time engaging in leisure-time physical activities than the physically active leisure group. Implications of these findings to assumptions related to the activity levels of older sport participants, suggestions for future research, and considerations for sport-related interventions aimed at enhancing health in older adulthood are discussed.

## 1. Introduction

Declining fertility rates and increasing life expectancy in countries around the world have resulted in an unprecedented demographic shift, in which older adults account for a significantly larger portion of the overall population [1],[2]. In Canada, for example, 16.1 percent of the population was 65 years of age or older in 2015. This number is expected to increase to 20.1 percent by 2024 [3]. Given the prevalence of chronic illness and disability in ageing populations, concerns regarding health-care costs and available resources continue to be raised [4],[5] highlighting the growing need for interventions involving environmental as well as behavioural strategies [6]. Physical activity (PA) is a modifiable lifestyle factor that has been cited as one of the most important preventative measures to reduce the morbidities often associated with older adulthood and to facilitate healthy physical and psychosocial ageing [7]. While the benefits of PA in later life are well established [7],[8], the combined effect of PA and sedentary behaviour (SB) on health is not fully understood [9]. SB is defined as “any waking behaviour characterized by an energy expenditure ≤ 1.5 METs while in a sitting or reclining posture” [10] and is distinguished as a separate construct from PA since it is possible for individuals to be classified as sufficiently active, but spend the majority of waking hours involved in sedentary activities, such as commuting, sitting at work, and/or during leisure time [10]–[14]. While some researchers have argued that high levels of SB are associated with adverse health outcomes independent of engagement in moderate to vigorous PA [10],[13],[15],[16], emerging evidence has indicated that participation in high levels of moderate to vigorous PA may eliminate or attenuate the deleterious effects of high SB [9],[17],[18]. Considering people engage in some level of PA and SB every day, additional research exploring both PA and SB patterns has been advocated [18].

Sedentarism in older adulthood is of particular concern for healthcare professionals, researchers, and policymakers. In comparison to the general population, older adults accumulate the highest level of sedentary time [12],[19], with reports of daily sedentary activity ranging from 5.3 to 12.0 hours [19]. Older adults spend most of their day in a sitting position for a number of reasons including pain or disease management, functional limitations, energy conservation, and lack of confidence or motivation [20]. Sociocultural pressures to rest and relax and perceptions that sitting is a “well-earned right” also prevail among older people [20]. Furthermore, the nature of activities geared towards older cohorts tend to revolve around sitting and are not perceived as harmful because they involve social and cognitive elements [20] or offer meaning and structure to one's daily life [21].

The pervasiveness of SB among ageing populations is alarming since increased sedentary time is associated with a multitude of negative health outcomes, even after accounting for participation in moderate-vigorous PA. The ill-effects of sedentarism in later life include increased all-cause mortality and frailty, reductions in self-reported health and the ability to perform activities of daily living, as well as, increased likelihood of overweight/obesity and related complications [12],[22]–[24]. In addition, greater SB in older people is a risk factor for cognitive decline (e.g., dementia) and cardiovascular health outcomes (e.g., hypertension) [25],[26], and displaces time that could be spent engaging in and reaping the health benefits of physical activity [13],[27].

To reduce SB in older adults, a better understanding of the reasons and conditions that influence this behaviour is needed [28]. Researchers have recognized the challenge of designing interventions and tailoring public health campaigns specific to SB in older people and have called for future studies on individual and contextual determinants of SB in this population [28]–[31]. One factor that may influence SB is PA involvement. The literature has justifiably focused on the duration and intensity of PA in relation to health outcomes of SB, however, additional knowledge could be gained by exploring the influence of *type* and *level* of PA involvement on participants' PA and SB patterns.

Sport, in particular, has been promoted in research and policy as a way to encourage healthy, active lifestyles in older adulthood [32],[33]. To date, the association between sport involvement later in life and SB has yet to be explored. Researchers have argued that SB decreases time spent in PA [13],[27] but recent evidence has suggested that SB and PA are distinct behaviours that occur independently [34],[35]. Studies have found younger, highly active athletes spend a high percentage of waking time outside of sport engaged in sedentary activities and that leisure-time SB among this population is not affected by time spent in high levels of aerobic training for sport [34]–[36]. However, there are considerable differences between younger, elite participants and older athletes, and as a result, it is unknown whether such findings apply to older people who participate in sport. The type and level of PA involvement may influence lifestyle choices, including time in sedentary activities. It has been suggested that sport involvement in older adulthood is associated with distinct outcomes that occur above and beyond those gained from participating in general leisure-time physical activity (LTPA) [37],[38]. More specifically, a systematic review on the benefits and costs of sport participation in older adulthood reported that older athletes may be more inclined to engage in health promoting behaviour such as cardiovascular and resistance training; although, it was unclear whether such behaviour was unique to sport in comparison to other forms of LTPA [39]. It is possible that the competitive nature of sport and adaptive motivation towards PA observed in elite older athletes [37] increase the likelihood that older sport participants engage in higher levels of LTPA and, in turn, are less likely to lead a predominantly sedentary lifestyle. However, comparisons of older adults across the PA continuum from inactive to competitive sport participants are needed to gain insight into whether type of PA involvement influences time spent in LTPA and SB. This information will inform public health promotion efforts as well as sport-related physical activity interventions for older adults.

Given that little is known about older sport participants' use of time in comparison to their leisurely active or inactive peers and whether the type of activity involvement is associated with differences in older adults' behaviour patterns, the purpose of the present study was to investigate the association between type of physical activity involvement in older adulthood and time spent in both LTPA and SB. To explore this association, the duration of time spent engaging in LTPA and SB throughout a given day was compared in a national sample of older adults (aged 65 years and older). More specifically, we considered time spent in LTPA and sedentary pursuits across four physical activity groups: competitive sport, non-competitive sport, physically active leisure, and inactivity. Given the lack of research exploring the influence of older adults' physical activity involvement on their use of time, our analyses were largely exploratory, although we hypothesized that engagement in LTPA and SB would be predicted by the presumed intensity of the activity group with competitive sport participants performing the largest amount of LTPA and the least amount of sedentary activity, followed by the non-competitive sport group, the physically active leisure group and finally, the inactive group.

## 2. Data and Methods

Data from Cycle 24 of the 2010 Statistics Canada's General Social Survey on Time Use (GSS-TU) were used for analyses in this study. The GSS-TU is a time-diary survey, which assesses self-reported involvement in 266 activities (including the type, duration, location, and who the respondent was with) over a 24-hour period. Additional data were collected within the survey pertaining to demographic information, perception of time, and subjective dimensions of well-being. Random-digit dialing of Canadian households (excluding Canadian territories and full-time residents of institutions) was conducted from January to December 2010. Following contact with a household, one respondent age 15 years or older was randomly selected to complete the survey in the official language of his/her choice and voluntary consent to participate was implied by the respondent's willingness to answer survey questions. Computer-assisted telephone interviewing automatically followed the questionnaire guide and allowed responses to be coded into existing or new categories as the interview progressed [40].

The GSS-TU has been distinguished as “one of the largest and most sophisticated [time-diary surveys] in the world” [41] as well as “the most comprehensive source of information about activity engagement among Canadians” [42]. Time-diary surveys, including the GSS-TU, are favoured by researchers interested in active living because the start and end times of all activities an individual engages in over the course of a day are recorded consecutively, and therefore, subjective accounts of activity behaviour are less susceptible to the effects of recall and social desirability biases [41]–[45]. In addition, detailed information collected on activity engagement provides an opportunity to assess PA and SB activities individually and simultaneously (e.g., sport participation or television viewing) and across various domains (e.g., leisure or occupation) [45]. A limitation of time-diary surveys is the relative lack of information regarding the intensity or effort level associated with each activity assessed. Such information is needed to classify activity on the basis of energy expenditures (e.g., low, light, moderate, and/or vigorous effort) [42],[43].

To address this shortcoming, Spinney and colleagues [44] harmonized the coding schemes of the GSS-TU with those listed in the Compendium of Physical Activities (CPA) Tracking Guide [46],[47]. The CPA enables the coding of PA assessed in records, logs, and surveys by assigning a specific metabolic equivalent of task (MET) intensity level to a wide range of activities [47]. A MET is defined as the “ratio of work metabolic rate to a standard resting metabolic rate” and represents the energy expenditure (measured in kcal/kg/hour) associated with a given activity [47]. Spinney et al. [44] reviewed the descriptions, examples, and exceptions for every activity assessed by the GSS-TU, identified potentially relevant CPA codes for each GSS-TU activity, and then calculated the median MET value of relevant CPA codes to assign a MET value to each GSS-TU activity (see Spinney et al. [44] for detailed information regarding the method of harmonizing CPA and GSS-TU coding schemes).

### 2.1. 2010 GSS-TU Sub-sample

Overall, the 2010 GSS-TU sample consisted of 15, 390 respondents, representing a response rate of 55.2 percent [40]. The present study was restricted to adults aged 65 years or older who provided responses to the sport participation activities (SPA) module of the GSS-TU, resulting in 1,760 cases for analysis.^1^ This sub-sample of independently living, older respondents was further grouped into LTPA involvement categories (i.e., competitive sport, non-competitive sport, physically active leisure, and inactive) based on their answers to SPA module questions: “Did you regularly participate in any sports during the past 12 months?” and “Did you participate in any competitions or tournaments in the last 12 months?”. Prior to beginning the SPA module, sport was clearly defined by the interviewer as: “. . . activities which involve training or competition with some level of physical intensity or organization. Leisure activities such as dance, fitness, fishing, or hiking, are not considered sport” [48]. Instructions for interviewer coding also acknowledged that “participate means as an athlete/participant – not as a coach, official or administrator; regularly means at least once a week during the season or for a certain period of the year” [48]. Respondents who indicated involvement in physical activities that could be considered sport in the time diary section of the GSS-TU (e.g., swimming, running, bicycling) but also answered “no” to the question regarding sport participation in the sports module were removed from the dataset (n = 37). This resulted in a final sub-sample size of 1,723.

### 2.2. Measurements

***SB.*** GSS-TU activities were classified as “low effort” (i.e., sedentary) if the MET value of the activity was equal to or less than 1.5. Examples of low effort activities include: meals/snacks, reading, socializing, watching TV, computer use, passive travel, and sleep. Total waking SB time was calculated by subtracting the reported duration of minutes spent in sleeping/napping from the sum of time spent (in minutes) in all GSS-TU activities meeting the low effort inclusion criteria.

***LTPA.*** This variable was calculated by summing the duration of time respondents reported engaging in LTPA, including: football, field hockey, baseball/softball, soccer, volleyball, hockey, basketball, tennis, squash/racquetball/paddleball, golf, miniature golf, swimming, waterskiing, ice skating, downhill skiing/snowboarding, skiing/sledding/curling, bowling, pool/ping-pong, pinball, home exercises, weight training, exercise class/aerobics, yoga, judo/boxing/wrestling/fencing, rowing/canoeing/kayaking, wind surfing/sailing, in-line skating/rollerblading, other sports (frisbee/catch/track and field/skateboarding), hunting (for leisure), fishing (for leisure), boating, camping, horseback riding/rodeo/jumping/dressage, other outdoor activities/excursions, walking, jogging/running, hiking, and bicycling.

***Covariates.*** Previous work has indicated that sociodemographic factors such as age, sex, and level of education influence participation in LTPA and sport [32], [49]–[51] as well as SB [52]. The 2010 GSS-TU sub-sample of older adults included four age groups: “65 to 69”, “70 to 74”, “75 to 79”, and “80 years and over”. Level of education was classified into five groups: “university degree” (includes doctorate, masters, and bachelor degrees), “college degree” (includes diploma or certificate from community college, trade, or technical school), “some university or college experience” (for those who did not complete their studies), “high school diploma”, and “less than high school” (indicates some secondary or elementary schooling or no schooling). Pre-analysis screening of the day designated for the interview (dichotomized as weekday or weekend) indicated an association with time spent in sedentary activities. These variables were adjusted for as covariates in the analyses to control for their potential influence on time spent in LTPA and SB.

### 2.3. Data analyses

Two analyses of covariance (ANCOVA) compared the total duration of minutes during a given day that were spent engaging in LTPA and low effort activities (i.e., SB) among PA involvement groups (i.e., competitive sport, non-competitive sport, physically active leisure, and inactive) while controlling for age, sex, level of education, and day of the interview. IBM SPSS Statistics 24 was used to conduct all statistical analyses.

## 3. Results

The largest group of respondents were represented in the “65 to 69” years age group (31.6%) and “less than high school” education category (31.6%). Most respondents identified as female (60.1%), were retired (74.5%), and currently married (46.5%) (see Table 1 for a description of sample characteristics). Competitive sport participants were involved in golf (n = 48), bowling (n = 16), curling (n = 11), ice hockey (n = 3), other (n = 3), swimming (n = 2), badminton (n = 1), downhill skiing (n = 1), tennis (n = 1), sailing/yachting (n = 1), and squash (n = 1). Non-competitive sport participants reported involvement in golf (n = 73), bowling (n = 29), swimming (n = 21), curling (n = 18), downhill skiing (n = 11), tennis (n = 11), cross country/nordic skiing (n = 6), cycling (n = 6), badminton (n = 3), canoeing/kayaking (n = 3), sailing/yachting (n = 3), figure skating (n = 2), ice hockey (n = 1), and softball (n = 1). Of the 245 competitive and non-competitive sport participants, 195 indicated involvement in only one sport, while 40 reported participating in two sports, and 10 reported involvement in three sports.

After controlling for age, sex, level of education, and designated day of the interview, a significant effect was found for type of PA involvement on time spent in SB, *F*(3, 1697) = 3.24, *p* = .021, ηp^2^ = .006 (see Table 2). Post-hoc analyses using Tukey's HSD indicated significant differences at *p* < .05 between competitive sport and physically active leisure (*p* = .035) and between competitive sport and inactive groups (*p* = .004). Estimated marginal means showed that respondents who participated in competitive sport spent the least number of minutes in SB (*M* = 492.00, *SE* = 22.64, 95% CI [447.61, 536.41]) in comparison to those who participated in physically active leisure (*M* = 544.79, *SE* = 10.60, 95% CI [523.99, 565.58]) and inactive respondents (*M* = 559.59, *SE* = 5.90, 95% CI [548.01, 571.16]). Levene's test indicated unequal variances (*F* = 3.50, *p* = .014).^2^

**Table 1.** Demographic Characteristics of the 2010 GSS-TU Older Respondents Sub-sample

	Competitive Sport		Non-competitive Sport		Physically Active Leisure		Inactive		Total	
Characteristic	n	%	n	%	n	%	n	%	n	%
Overall	76	4.4	169	9.8	347	20.1	1131	65.6	1723	100
Age										
65 to 69 years	33	43.4	71	42.0	124	35.7	317	28.0	545	31.6
70 to 74 years	20	26.3	35	20.7	79	22.8	275	24.3	409	23.7
75 to 79 years	15	19.7	31	18.3	58	16.7	232	20.5	336	19.5
80+ years	8	10.5	32	18.9	86	24.8	307	27.1	433	25.1
Sex										
Male	45	59.2	92	54.4	142	40.9	409	36.2	688	39.9
Female	31	40.8	77	45.6	205	59.1	722	63.8	1035	60.1
Education										
University degree	22	28.9	43	25.4	79	22.8	165	14.6	309	17.9
College degree	17	22.4	42	24.9	85	24.5	279	24.7	423	24.6
Some university or college experience	9	11.8	27	16.0	43	12.4	123	10.9	202	11.7
High school diploma	10	13.2	27	16.0	40	11.5	150	13.3	227	13.2
Less than high school	18	23.7	29	17.2	97	28.0	400	35.4	544	31.6
Missing response	0	0.0	1	0.6	3	0.9	14	1.2	18	1.0
Main Activity										
Working	11	14.5	20	11.8	29	8.4	103	9.1	163	9.5
Looking for work	0	0.0	2	1.2	0	0.0	3	0.3	5	0.3
Caring for children	0	0.0	0	0.0	0	0.0	10	0.9	10	0.6
Household work	4	5.3	11	6.5	29	8.4	114	10.1	158	9.2
Retired	57	75.0	129	76.3	269	77.5	828	73.2	1283	74.5
Long term illness	0	0.0	0	0.0	6	1.7	12	1.1	18	1.0
Volunteering/caregiving	2	2.6	4	2.4	10	2.9	33	2.9	49	2.8
Other	2	2.6	2	1.2	1	0.3	19	1.7	24	1.4
Missing response	0	0.0	1	0.6	3	0.9	9	0.8	13	0.7
Marital Status										
Married	53	69.7	101	59.8	161	46.4	503	44.5	818	47.5
Common-law	0	0.0	3	1.8	10	2.9	19	1.7	32	1.9
Widowed	13	17.1	37	21.9	118	34.0	403	35.6	571	33.1
Separated	2	2.6	2	1.2	6	1.7	15	1.3	25	1.5
Divorced	6	7.9	13	7.7	30	8.6	112	9.9	161	9.3
Never married	2	2.6	12	7.1	21	6.0	75	6.6	110	6.4
Missing response	0	0.0	1	0.6	1	0.3	4	0.4	6	0.3

**Table 2.** ANCOVA and Post-hoc Tests for Time Spent in SB Across LTPA Groups

	LTPA Groups				ANCOVA Statistics		
**Variable**	Competitive Sport (1)	Non-competitive Sport (2)	Physically Active Leisure (3)	Inactive (4)	*F*-statistic (3, 1697)	Tukey post-hoc test	*p*-value
Mean (SE)	492.0 (22.6)	538.6 (15.3)	544.8 (10.6)	559.6 (5.9)	3.24*	1 vs. 2	0.09
						1 vs. 3	0.04*
						1 vs. 4	0.00*
						2 vs. 3	0.74
						2 vs. 4	0.20
						3 vs. 4	0.22

Notes: *significant at *p*≤ 0.05; LTPA = Leisure-time physical activity; SE = standard error

Controlling for age, sex, and level of education, a significant effect was also found for PA group and time spent in LTPA, *F*(3, 1698) = 253.87, *p* < .001, ηp^2^ = .312 (see Table 3). Tukey's HSD post hoc tests revealed significant differences at *p* < .05 between competitive sport and physically active leisure groups (*p* < .001), competitive sport and inactive groups (*p* < .001), non-competitive sport and physically active leisure groups (*p* < .001), non-competitive sport and inactive groups (*p* < .001), and physically active leisure and inactive groups (*p* < .001). Estimated marginal means indicated that physically active leisure participants spent the most minutes in LTPA (*M* = 72.78, *SE* = 2.46, 95% CI [67.96, 77.60]), followed by non-competitive sport participants (*M* = 54.67, *SE* = 3.54, 95% CI [47.73, 61.61]), competitive sport participants (*M* = 47.20, *SE* = 5.25, 95% CI [36.90, 57.50]), and inactive respondents (*M* = 0.81, *SE* = 1.37, 95% CI [−1.88, 3.49]). Levene's test indicated unequal variances (*F* = 321.54, *p* < .001).^3^

**Table 3.** ANCOVA and Post-hoc Tests for Time Spent in LTPA Across LTPA Groups

	LTPA Groups				ANCOVA Statistics		
**Variable**	Competitive Sport (1)	Non-competitive Sport (2)	Physically Active Leisure (3)	Inactive (4)	*F*-statistic (3, 1698)	Tukey post-hoc test	*p*-value
Mean (SE)	47.2 (5.3)	54.7 (3.5)	72.8 (2.5)	0.8 (1.4)	.000*	1 vs. 2	0.24
						1 vs. 3	0.00*
						1 vs. 4	0.00*
						2 vs. 3	0.00*
						2 vs. 4	0.00*
						3 vs. 4	0.00*

Notes: *significant at *p*≤0.05; LTPA = Leisure-time physical activity; SE = standard error

## 4. Discussion

The present study examined the influence of type of PA involvement (competitive sport vs. non-competitive sport vs. physically active leisure vs. inactivity) on time spent in SB and LTPA. Results indicated that respondents involved in competitive sport reported significantly fewer minutes of SB in a given day than those involved in general forms of LTPA or those categorized as inactive. Competitive sport participants spent an average of 492 minutes (8.2 hours) engaging in sedentary activities, while physically active leisure participants spent an average of 545 minutes (9.1 hours) and inactive respondents spent an average of 560 minutes (9.3 hours) engaging in SB. These findings are within the range of SB time reported in the literature [19] and are comparable to a recent Canadian study indicating older adults (aged 60–79 years) averaged 600 minutes of SB during the day [53].

Given the adverse health outcomes associated with sedentary lifestyles and the prevalence of SB in older age, the lower level of SB in older competitive sport participants is noteworthy. However, the practical significance of lower levels of SB is less evident. While overall reductions in sedentary time have been advocated to prevent and/or ameliorate the deleterious effects of prolonged SB, the extent to which older adults must reduce daily SB to reap physical and psychosocial health benefits has yet to be established [54],[55]. Studies have found the risk of all-cause mortality is reduced when older adults spend less than 8 hours of the day sitting and that this risk increases with every additional hour individuals spend sitting per day [56],[57]. Another study on SB in older adults found physical function (i.e., balance, strength, walking time) decreased per hour increase in SB [55]. Future research should clarify the dose-response relationship between small reductions in overall SB time and health outcomes in older populations.

Further, SB has been identified as “complex and multi-faceted” [58]. Researchers have recognized that the type or mode of SB, frequency of SB bouts, and number of interruptions to SB should be studied in addition to overall sedentary time [14],[19]. For instance, sedentary activities classified as social or cognitive have been associated with adaptive health outcomes [6],[11],[24],[59],[60] and breaks in prolonged SB are associated with health benefits independent of overall reductions in SB time [13],[61],[62]. Domain-specific SB (e.g., leisure, occupational SB) and the sequence or timing of SB during the day were not examined in the present study and should be included in future work on SB in older competitive sport participants to gain a better understanding of the context and pattern of SB within this unique population. Qualitative research is also recommended to gain insight into older sport participants' understanding, motivation, and views of SB. This information will inform the development of sport-based health interventions and/or community programs that can influence SB in older adults.

The results of the present study also suggest competition may be a key feature of sport participation later in life. Significant group differences in SB time were found for the competitive participants but not in the non-competitive sport group, indicating that the amount of leisure time spent in SB is not the same for all older adults involved in sport. However, the cross-sectional nature of this study makes it difficult to determine whether older people who spend less time in sedentary pursuits tend to gravitate to competitive forms of sport or whether competitive sport participation encourages less time in SB per day. Nor is it clear why competitive sport participation in later life may influence SB. Langley and Knight [63] outlined the efforts of one competitive older athlete to enhance his success in sport by “taking care for his body”. In this case, the athlete engaged in health promoting behaviour including cardiovascular and strength training to improve his ability to be successful in sport. It may be that older competitive sport participants are more aware of the ill effects of sedentarism and make a conscious effort to reduce SB within their daily schedule. Since overall time spent in training for sport was not assessed in this study, the influence of physical efforts to prepare for competition on SB in older competitive sport participants is not known. Additional research is recommended to fully understand the reasoning behind older competitive sport participants' use of time.

A limitation of the GSS-TU is the lack of data pertaining to the intensity, frequency, and duration of sport participation over time. Although researchers have acknowledged that competitive sport motivates older adults to push their bodies to their limits [64], differences in the intensity and frequency of sport participation between competitive and non-competitive sport groups cannot be discerned from the information provided in the GSS-TU. Moreover, respondents were classified into the competitive sport group on the basis of a single question that assessed participation in a tournament or competition within the past 12 months. Many older adults are motivated to compete for reasons that extend beyond extrinsic rewards such as winning, including travel, fun, enjoyment, social interaction, creative expression, and improving/maintaining physical health [38],[49],[65].

The work of Dionigi [32] has also highlighted that: “… nowadays sport is highly valued across the lifespan and is used as a tool in health promotion policy and practice to encourage older people to remain active…”. Our findings challenge current assumptions that older competitive sport participants are the “physically elite” of ageing populations distinguished from their generally active peers for their regular participation in intense exercise training and high level of physical functioning [66]. Older competitive and non-competitive sport participants spent significantly less time involved in LTPA than their leisurely active peers. Average time spent in LTPA in a given day was 72.8 minutes for the physically active leisure group, 54.7 minutes for the non-competitive sport participants, and 47.2 minutes for the competitive sport group. It is encouraging that the competitive and non-competitive sport sub-sample of older adults spent more time in LTPA than self-reported LTPA of the general Canadian population aged 60 to 79 [67]. Yet, the results raise important questions about the types of LTPA that should be encouraged to promote a physically active lifestyle in later life. Although older athletes are upheld in the literature as role models of active living and successful ageing [37] and researchers have suggested that the pursuit of athletic goals encourages older people to be fit, healthy, and put forth greater effort [38],[50],[68],[69], the contribution of sport involvement in later life to individual and community health in comparison to other forms of LTPA has been debated [70]. Research has indicated that sport does not necessarily increase LTPA time in older adults. Shaulis, Golding, and Tandy [71] found no difference in functional fitness, modes of exercise, or training frequencies of older athletes and non-athletes and noted that some athletes engaged in little to no training for competition. It is clear that the relationship between competition and training/PA in the older adult population is not simple.

While mainstream sport is understood in terms of performance enhancement [32], sport participation in older adulthood is often constrained by age-related stereotypes and sociocultural norms regarding the “appropriateness” of vigorous, high intensity, competitive physical activity in older adulthood [49],[64],[72]. The influence of sport participation on LTPA in later life may be dependent on the goals of the sport program, desires of participants, and societal expectations. Future work should explore older sport participants' views on LTPA and perceptions of sport as a tool for LTPA and exercise promotion. Furthermore, the intended outcomes of sport for older people should be carefully contemplated in the design and structure of sport-based health programming in later life and older people should be consulted throughout the development and implementation of these programs.

It is important to note that the GSS-TU examined activity involvement over the course of a given day. It is possible that respondents were more or less active on days of the week not captured by the GSS-TU and that participants' recollection of daily activities was subject to social desirability bias and recall error characteristic of self-report measures [73],[74]. Studies on athletic, physically active, and inactive older adults that use objective measures of LTPA in conjunction with self-report measures are recommended to gather a more holistic picture of behaviour patterns across ageing populations over longer periods of time. Researchers have also acknowledged the challenges associated with calculating exact MET values based on individual differences, particularly in relation to age [44],[47]. Hall et al. [75] noted that in comparison to younger adults, the energy costs associated with completing daily tasks is higher in older adults. However, this may not be a predominant concern in the present study because estimates of MET values were only calculated for activities involving low effort. Finally, the length of the survey has been cited as burdensome for respondents, which could affect the quality of data collected [42].

## 5. Conclusion

This study highlights the need to re-examine assumptions related to the influence of sport involvement in older adulthood on participants' use of leisure time. While sport-related programs undoubtedly offer a wide range of benefits for older people, the results of this study suggest that sport participation and LTPA may not be strongly related. Research and policy have emphasized the use of sport as a tool for promoting health in the latter stages of life [32],[33], but our work illustrates the complexity of sport participation in older adulthood and raises questions about the types of LTPA that should be encouraged to promote physically active lifestyles among ageing populations. Before efforts to change older adults' use of time can be designed and implemented, research is needed to determine whether the differences in time-use among the PA groups affects older adults' health and whether sport enhances health outcomes through its influence on SB. It should also be noted that notions of “health” have different meanings for different individuals. While researchers, clinicians, and policymakers continue to emphasize the importance of a physically active and less sedentary lifestyle to healthy ageing, a critical approach to sport-based health research and programming should consider the perspectives and experiences of older adults in order to inform the development of meaningful sport-based interventions across ageing populations, which may extend beyond functional and physical health outcomes.

## Footnotes

^1^ GSS-TU respondents were randomly divided into two sub-samples. Approximately half of the sample completed the Cultural Activities module, while the other half completed the SPA module [40].

^2^ While significant results of the Levene's test indicate the assumption of variance homogeneity may have been violated, a Kruskal-Wallis test was conducted in parallel and found a significant effect for physical activity group and time spent in SB, *H*(3) = 13.72, *p* = .003.

^3^ While significant results of the Levene's test indicate the assumption of variance homogeneity may have been violated, a Kruskal-Wallis test was conducted in parallel and found a significant effect for physical activity group and time spent in LTPA, *H*(3) = 1327, *p* < .001.
